# Spatial Instability during Precision Grip–Lift in Children with Poor Manual Dexterity

**DOI:** 10.3390/brainsci12050598

**Published:** 2022-05-04

**Authors:** Yuki Nishi, Satoshi Nobusako, Taeko Tsujimoto, Ayami Sakai, Akio Nakai, Shu Morioka

**Affiliations:** 1 Neurorehabilitation Research Center, Kio University, Koryo, Kitatkatsuragi-gun, Nara 635-0832, Japan; s.nobusako@kio.ac.jp (S.N.); s.morioka@kio.ac.jp (S.M.); 2 Department of Rehabilitation Medicine, Kanmaki, Kitatkatsuragi-gun, Nishiyamato Rehabilitation Hospital, Nara 639-0218, Japan; 3 Graduate School of Health Science, Koryo, Kitatkatsuragi-gun, Kio University, Nara 635-0832, Japan; 4 Department of Rehabilitation, Nishide Clinic, Kashiwara, Osaka 532-0002, Japan; kwpcw480@yahoo.co.jp; 5 Department of Rehabilitation, Higashi Osaka Yamaji Hospital, HigashiOsaka, Osaka 578-0925, Japan; s.a.19900426@gmail.com; 6 Graduate School of Clinical Education & The Center for the Study of Child Development, Institute for Education, Mukogawa Women’s University, Nishinomiya, Hyogo 663-8558, Japan; anakai.kodomo@gmail.com

**Keywords:** grip force, spatial instability, variability, motor clumsiness

## Abstract

Although children with developmental coordination disorder (DCD) show impaired precision grip control due to a sensory-motor integration deficit, their spatial instability (such as changes in force direction and object roll during a precision grip task) is unclear. Herein, we investigated the spatial instability in the precision grip force control of children with poor manual dexterity. We divided 66 school-aged children who performed a precision gripping and lifting of heavy- or lightweight objects into those with low manual dexterity (*n* = 11) and those with high manual dexterity (*n* = 55) as revealed by the Movement Assessment Battery for Children (2nd edition). The group and weight effects were then determined. The results revealed that the total trajectory lengths of the center of pressure (COP) were longer in the lightweight object data of the children in the low-manual-dexterity group and were related to the children’s grip force. The low-manual-dexterity group also showed a shifted COP position from the center of the object in the medial–lateral direction and in the object roll regardless of the object’s weight; these were closely related in both weights’ tests. These results demonstrated that children with poor manual dexterity show spatial instability and different adaptations to the weight of objects during a precision grip task. Further studies are needed to determine whether these findings would be replicated in children with a diagnosis of DCD.

## 1. Introduction

Children with developmental coordination disorder (DCD) are often given the label “motor clumsiness” and account for 5–6% of the school-aged child population [1,2]. DCD is characterized by difficulties of fine and gross motor coordination and a deficit of motor learning [3,4]. A systematic review and meta-analysis documented that one of the common coordinated motor problems of children with DCD is the impaired control of precision grip–lift force control between the index finger and the thumb for holding an object [5,6]. Children with DCD also show impairments in activities of daily living for which precision grip controls are crucial, such as writing and sports that involve a ball. Since these motor control deficits persist into adolescence and adulthood, DCD is described as a chronic disability [2,7,8]. The impairment of the precision grip in children with DCD is a clinical problem that thus requires greater attention.

Generally, when a person grasps and lifts an object with a symmetrical mass in a precision grip, the grasping position must be closer to the center of the horizontal plane of the object in order to minimize the object’s roll, and sufficient grip force must be exerted to prevent slipping of the object [9,10,11]. When objects with an identical visual appearance but differing weights are to be lifted, grip and lift forces need to be adapted based on the error information between the sensory feedback and an erroneous programming of force [11]. These sophisticated grip controls provided by the sensory-motor integration in the internal model allow for complex daily activities [12].

In a task that requires a precision grip, children with DCD showed larger variability of grip force and high safety margins of grip force compared to typically developing children [13,14]. It was also reported that in a reaching movement task, children with DCD exhibited larger variability of grip force and increased movement error [15,16]. These motor impairments in children with DCD are often explained as an internal model deficit that reduces both their predictive motor control and subsequent online correction [5]. In addition, children with low manual dexterity without a diagnosis of DCD have also been shown to have difficulty with predictive motor control and online correction in the internal model [17,18,19]. Based on the above-cited findings, we speculated that in children with low manual dexterity, there may be a shift of the grasping position of an object due to reaching movement errors and/or spatial instability (such as unwanted object rotation or finger slipping). In addition, children with low manual dexterity may not have sufficiently adaptive force control over the different weights of objects, but this has not been evaluated. It is known that spatial instabilities of a precision grip can be described using the tilt angle of an object measured by accelerometers and the trajectory of the center of pressure (COP) of the grip [20,21].

In this study, we examined (i) the mean and the coefficient of variation of grip force as parameters of precise grip force control, (ii) the mean COP position as the gripping position, (iii) the total trajectory length of the COP, and (iv) the mean degree of object rolls as a parameter of spatial stability during the grasping and lifting of freely movable objects in children with low or high manual dexterity. We also assessed how these parameters vary with the object’s weight. We hypothesized that children with low manual dexterity would show high variability of grip force and high spatial instability because in children with low manual dexterity, precision grip controls may be impaired due to a reduced ability to effectively use feedback information for movement and/or due to a reduced ability to correct movements in real time [16,22,23].

## 2. Materials and Methods

### 2.1. Participants

Children with typical development who were enrolled in regular classes at public primary schools in Nara and Osaka, Japan, were recruited. A total of 66 school-age children (mean age  ±  standard deviation, age 9.2  ±  2.0 years, range 6–12 years; 34 boys and 32 girls; 63 right-handed) were enrolled. The exclusion criteria were: (1) a general medical condition (e.g., cerebral palsy, hemiplegia, and muscular dystrophy), (2) diagnosis of a developmental disorder (e.g., autism spectrum disorder, attention-deficit hyperactivity disorder, and learning disorder), and (3) diagnosis of intellectual disability. Although DCD was not an exclusion criterion, none of the children in the study had a diagnosis of DCD. Eligibility was confirmed through an interview of the children’s parents and the results of regular checkups, which were provided by the school doctor at each school. All experimental procedures were approved by the local ethics committee of the Graduate School and Faculty of Health Sciences at Kio University (approval no. H27-33). The study posed no foreseeable risks, and no personally identifying information was collected. The participants (children and their parents) provided background information and written informed consent. The study procedures complied with the ethical standards of the 1964 Declaration of Helsinki regarding the treatment of human participants in research.

### 2.2. Experimental Procedures

The children underwent the manual dexterity test of the Movement Assessment Battery for Children—2nd Edition (M-ABC2) and the grip–lift task. The manual dexterity test of the M-ABC2 and grip–lift tasks were performed within 15 and 30 min, respectively, and the order in which the tasks were performed was randomized.

### 2.3. The M-ABC2 Manual Dexterity Test

For the investigation of the relationship between precision grip force controls and upper limb/hand coordination function, only the manual dexterity test of the M-ABC2 was evaluated. The manual dexterity test of the M-ABC2 [24] is a standardized, age-adjusted test used to identify motor problems in children, in which different tasks are administered to children in different age bands. The M-ABC2 has good test–retest reliability (the minimum value at any age was 0.75), inter-rater value (0.70), and concurrent validity [24]. It uses the following three age bands: 3–6, 7–10, and 11–16 years.

Our study included children aged 6–12 years. Each child took three tests that were appropriate for their age band. The 6-year-old children (*n* = 10; 5 male participants; 7 right-handed) were in age band 1 and were administered the following three tests: a posting coins test, threading beads test, and drawing trail I test. The 7- to 10-year-old children (*n* = 34; 19 male participants; 34 right-handed) were in age band 2 and were administered the placing pegs test, threading lace test, and drawing trail II test. The 11- and 12-year-old children (*n* = 22; 8 male participants; 22 right-handed) were in age band 3 and were administered the turning pegs test, a triangle with nuts and bolts test, and the drawing trail III test. The children’s standard scores were calculated from their raw scores, based on the examiner’s M-ABC2 manual. The standard score reflects the degree of manual dexterity for each year of age, in which a higher standard score represents better manual dexterity within the respective age group. A specifically trained and certified physical therapist administered all of these assessments.

We used the children’s M-ABC2 results to classify the children into the following two manual dexterity groups. Children in the 16th percentile (standard score, 7) or lower were classified as the low-manual-dexterity group, and the children in the 25th percentile (standard score, 8) or higher were classified as the high-manual-dexterity group.

### 2.4. Apparatus

**Figure 1:**
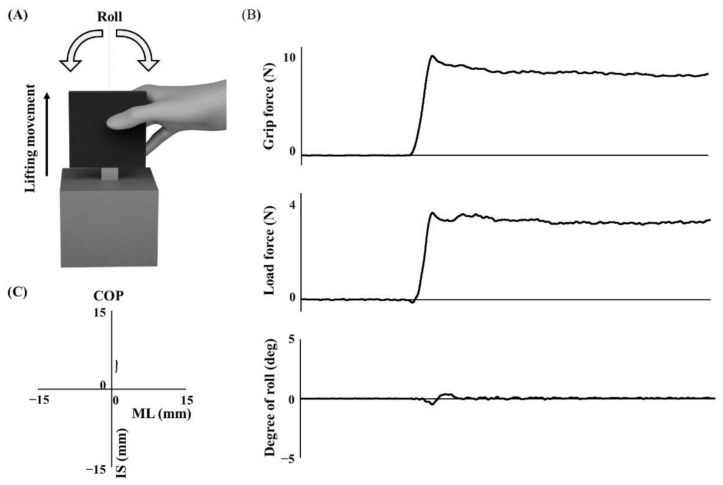
A depiction of the grip instrument and traces of sensor data. (**A**) The gripped black plate is the force/torque transducer, and the gray box is an opaque plastic box that can contain a weight (500 g). (**B**,**C**) Sensor data for 5 sec from lift-off. COP: center of pressure. ML: medial–lateral direction. IS: inferior–superior direction.

Each child grasped and lifted an iron six-component force/torque transducer (width 80 mm, height 80 mm, depth 11 mm, #M3D-EL-FP-U, Tec Gihan, Ibaraki, Japan) with an opaque plastic box mounted underneath (Figure 1A). The box was either empty or contained a 500 g weight which was securely fixed within the symmetry of the box. The apparatus’ center of mass was exactly vertical, and the symmetrical point of the apparatus prevented no torque by deviation of the center of mass. The total weight of the apparatus was 800 g when the box contained a 500 g weight (= the heavyweight condition) and 300 g when the box was empty (= the lightweight condition) [13]. The grip force (GF), load force (LF), the degree of roll calculated by the vertical accelerometer of the apparatus, and the center of pressure (COP) of the inferior–superior (I/S) and medial–lateral (M/L) directions were collected at a sampling rate of 100 Hz (Figure 1B,C). The GF is the internal force exerted by the fingers on the object, and the LF is the vertical inertia force generated when the object is moved up and down. The degree of roll indicates the object’s degree of rotation around its anterior–posterior axis.

### 2.5. Grip–Lift Task

The child sat on a chair facing a table, with their feet on the ground. The child’s dominant hand was placed on the table 15 cm away from them in the midsagittal plane. The instrumental object was placed on the table 15 cm from the child’s dominant hand (i.e., 30 cm from the child). The grip–lift test was explained to the child, who was then asked to lift the object to their shoulder using the precision grip between the dominant index finger and thumb, hold it for 5 s, and replace it on the table. Each child lifted the 800 g object in five trials as practice. Ten consecutive lifts with the 800 g weight (heavy condition) were then performed, followed by ten lifts with the 300 g object (light condition). The inter-trial interval between lifts was approximately 5 s. The insertion of the pre-calibrated weight into the experimental apparatus was carried out without the children’s knowledge or observation. The parameters of the last five trials performed with each weight were analyzed [13].

### 2.6. Data Processing and Statistical Analyses

All data processing related to the raw signals measured in the grip–lift task was performed using custom-made software designed on MATLAB R2021a (MathWorks, Natick, MA, USA). We calculated the following as the grip–lift parameters: the mean GF, the coefficient of variation (CV) of the grip force, the total trajectory length of the COP (COP trajectory), the mean COP position in inferior–superior (IS) and medial–lateral (ML) directions, and the mean degree of roll. The mean GF was calculated for 5 s from the lift-off of the object, which was defined as the time point at which the load force reached the gravitational force exerted on the object (i.e., when the lift force exceeded 98% of the object weight) [13,25]. A low mean GF according to the object’s weight indicates that the GF is adaptively adjusted to prevent the object from slipping.

The CV, which indicates the variability of grip force, was calculated as divided by the standard deviation of the mean grip force for 5 s from lift-off [26]. A high CV indicates high force variability. The total length of the COP is the total displacement induced by a deviated finger force direction, and thus, a low total length of the COP indicates high stability of the force [27,28]. The mean COP position was defined as the deviation of the grasping position from the center of the grasped object in both the inferior–superior (IS) and medial–lateral (ML) direction [20]. A mean COP of 0 thus indicates that the grasping position is at the center of the object. The total length of the COP and the mean COP were calculated for 5 s from lift-off. The mean degree of roll, which is defined as the degree of rotation around the anterior–posterior axis, was calculated as the mean absolute value of the angle between the acceleration of the vertical axis of the object and the gravity vector within the frontal plane of the object [29,30]. A value of 0 as the mean degree of roll indicates that there was no rotation of the object around the anterior–posterior axis during the lifting and gripping of the object.

We used the software program R (ver. 4.1.0) for all statistical analyses. We compared the age, sex, and preferred hand distribution between the low- and high-manual-dexterity groups using the *t* test and χ^2^ test, respectively. For the examination of the children’s precision grip control, we performed a 2 × 2 [Weight (heavy/light weight of the object)  ×  Group (high/low manual dexterity)] repeated measures analysis of variance (RM-ANOVA). Partial eta square (ηp^2^) values were calculated for the identification of effect sizes. Independent and dependent *t* tests were used for a post-hoc analysis when significant group × condition interactions were identified.

Independent *t* tests were used to compare demographics between groups, and Cohen’s d-values were calculated to indicate effect sizes. A commonly used interpretation is to refer to effect sizes as small (d  =  0.2; ηp^2^  =  0.01), medium (d  =  0.5; ηp^2^  =  0.06), and large (d  =  0.8; ηp^2^  =  0.14) [31]. We evaluated the associations between each variable of precision grip control and the M-ABC2 by determining the Spearman’s rank correlation coefficient. The analysis and a post-hoc analysis of two-way ANOVA were corrected for multiple comparisons using Holm corrections [32].

## 3. Results

The 66 children’s demographic characteristics are summarized in Table 1. There were 11 children (mean age  ±  standard deviation, 9.00  ±  2.30 years; range 6–12 years old; 7 girls, 4 boys; 10 right-handed) in the low-manual-dexterity group, i.e., their score on the M-ABC2 manual dexterity test was below the 16th percentile (standard score, 7). The other 55 children (mean age  ±  standard deviation, 9.16  ±  1.93 years old, range 6–12 years old; 27 girls, 28 boys; 53 right-handed) were in the high-manual-dexterity group, having scored above the 25th percentile on the M-ABC2 manual dexterity test (standard score, 8). There were no significant between-group differences in age (*p* = 0.82) *t* test, sex (χ^2^ test = 0.78, df  =  1, *p* = 0.38), or preferred hand (χ^2^ test = 0.63, df  =  1, *p* = 0.43).

Table 2 provides the F-values, *p*-values, and ηp^2^ values of the precision grip control variables in the RM-ANOVA for both groups. The RM-ANOVA of the mean GF revealed significant main effects for Weight, but not for Group. The interaction of Weight × Group was not significant. The RM-ANOVA of the CV of grip force revealed significant main effects for Weight and for Group, but not an interaction of Weight × Group.

The RM-ANOVA of the total trajectory length of the COP (COP trajectory) revealed significant main effects for both Weight and Group and revealed that the interaction of Weight  × Group was significant. Post-hoc analyses were performed for the COP trajectory, demonstrating that the COP trajectory for the heavyweight object was significantly higher than that for the lightweight object in both groups. In addition, the COP trajectory in the low-manual-dexterity group was significantly higher than that of the high-manual-dexterity group for the lightweight object but not the heavyweight object.

In the IS direction, the RM-ANOVA of the mean COP position revealed no significant main effects for Weight or Group. The interaction of Weight × Group was not significant. In the ML direction, the RM-ANOVA of the mean COP position revealed no significant main effects for Weight but showed significant main effects for Group. The interaction of Weight × Group was not significant. The RM-ANOVA of the mean degree of roll detected no significant main effects for Weight but revealed significant main effects for Group. The interaction of Weight  × Group was not significant.

**Figure 2:**
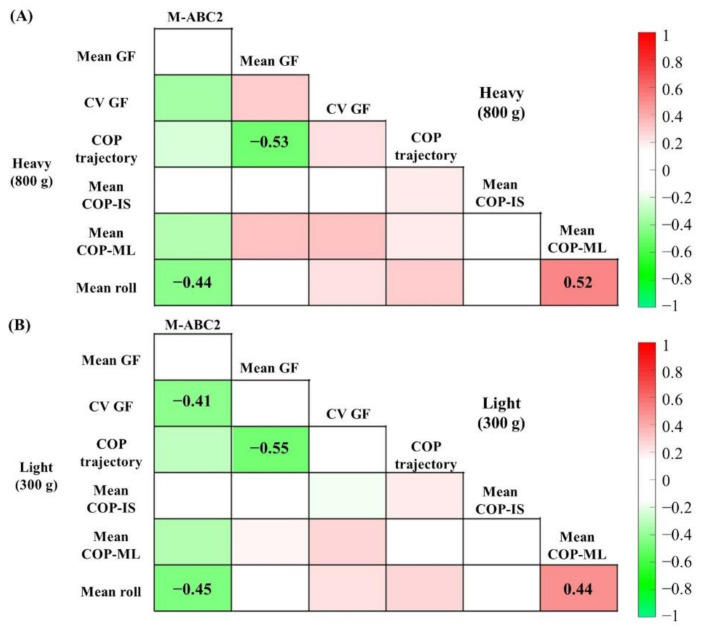
Heat map showing correlation coefficients in the (**A**) heavyweight and (**B**) lightweight object conditions. Darker pixels reflect higher correlation values (red: positive, green: negative). The r-value is indicated only in the pixels when the correlation was significant at *p* < 0.05 using Holm correction.

The associations among the children’s M-ABC2 results and precision grip control are presented in Figure 2. In the heavyweight condition, larger grip forces were associated with shorter COP trajectories, and larger mean roll was significantly correlated with a lower M-ABC2 and a higher mean COP-ML. In the lightweight condition, the higher CV was associated with lower M-ABC2, and larger grip forces were associated with shorter COP trajectories. The higher mean roll was also associated with both lower M-ABC2 and higher mean COP-ML.

## 4. Discussion

In this study, school-aged children performed precision gripping and lifting heavy- and lightweight objects, and we compared the children whose M-ABC2 results indicated low and high manual dexterity to their precision grip control data. Our results demonstrated that the total trajectory lengths of the COP (COP trajectory) were longer in the lightweight condition in the low-manual-dexterity group and were related to the grip force. The low-manual-dexterity group also showed a shifted COP position from the center of the object in the medial–lateral direction and object roll regardless of the object’s weight, and these data were closely related in both object-weight conditions. All of the results of the RM-ANOVAs with significant differences showed medium or large effect sizes. To the best of our knowledge, this is the first study in which the children with low manual dexterity showed spatial instabilities (i.e., COP trajectory and object roll) in the precision grip–lift task.

We observed that children with both low and high manual dexterity adapted grip force to the weights of the object during the precision grip–lift of the objects, and larger grip forces were associated with shorter COP trajectories regardless of the object’s weight. This finding is consistent with those of studies of adults [27,28]. A COP can be shifted by a deviated force direction during the lifting and holding of an object [33]. Our present study showed that in the heavy-object condition, the COP trajectory was shortened, and there was no significant difference between the results of the children with low and high manual dexterity.

Children with low or high manual dexterity might exert larger grip forces that increase stability because lifting and holding a heavy object requires high effort. On the other hand, the lightweight object used in this study requires a relatively low grip force for lifting and holding the object, and the children strategically prioritized energy conservation over spatial stability, which was universal regardless of their level of manual dexterity. Moreover, one of the key findings of this study is that the COP trajectory of the lightweight object in the children with low manual dexterity was greater than that in the children with high manual dexterity. Therefore, the COP trajectory may be affected by not only a strategy that prioritizes energy conservation over spatial stability but also motor clumsiness.

Children with DCD are known to have impaired integration of feedback information with the motor command in the internal model and to have reduced ability to effectively use tactile information for movement [22,23,34]. Therefore, in the present children with low manual dexterity, slipping, rolling, or twisting between the fingertip and the object were not explicitly perceived and not corrected, resulting in an increased COP trajectory. However, our results showed that the COP trajectory was not related to variability of the grip force, and thus, a deviated force direction may not necessarily correspond to variability of grip force [35].

Another key finding of our present investigation is that in the low-manual-dexterity group, the object roll occurred during lifting and holding regardless of the different object weights and was strongly related to the COP positions in the ML direction. In general, grip forces in parallel with digit placement are modulated according to an object’s center of mass, and in a case in which the center of mass is the center of the object, the gripping position is implicitly centered to prevent object roll [36]. In the present study, the object roll observed in the children with low manual dexterity can thus be attributed to the deviation of the gripping position from the center of the object and a loss of correction by grip force for torque. Considering the prior findings of reaching error in children with DCD [16,37], we speculate that the deviations of the gripping position may be caused by reaching error and lead to torque, resulting in object roll. This new insight regarding the deviation of gripping position in object roll should be considered in future research concerning the precision grip force controls of a freely movable object in children with DCD.

Moreover, a loss of correction by grip force for torque in an individual with low manual dexterity may occur due to impairment of online motor control by a sensory-motor integration deficit. A mismatch between motor predictions (e.g., grip force) and actual sensory feedback (e.g., tactile information of weight, friction and torque, and visual information of object slipping and roll) generates error signals, which modulate the unfolding motor commands in real time and update future motor prediction [38,39]. Therefore, their difficulty integrating the sensory feedback of the torque and/or object roll may prevent modulation of the object roll and gripping position [16,40].

Several study limitations must be noted. None of the children in the study, including those in the low-manual-dexterity group, had received a diagnosis of DCD. However, because this study focused on manual dexterity, we used only the M-ABC2 manual dexterity test component score. Interpretations of our findings should thus be limited to children with manual dexterity difficulties. We assumed that the impaired precision grip control in children with low manual dexterity has the same internal modeling deficits as low manual dexterity in DCD, but this cannot be determined from the present results. Further studies are needed to evaluate all subscale tasks of the M-ABC2 among children who fully meet the DSM-5 DCD diagnostic criteria A–D and have been diagnosed with DCD. The children in the study did not include children with attention-deficit/hyperactivity disorder (ADHD) or sensory disorders, but the degree of inattention, impulsivity/hyperactivity, and tactile sensitivity were not investigated. Reference [13] reported that changes in precision grip–lift control in children with attention deficit were similar to those in children with DCD, and the variability of the grip force was correlated with the hyperactivity score in the DSM-IV score. Therefore, ADHD traits should be measured in a future study to yield more definitive conclusions. In addition, in the present study, the children gripped a lightweight object after gripping a heavyweight object, and we analyzed the last five of ten trials of each weight to evaluate the precision grip controls in a stable and predictable condition [13]; however, in the children with low manual dexterity, the precision grip controls for the lightweight object may have been affected by the trials with the heavyweight object because a sensory-motor integration deficit makes motor adaptation to the different weights of an object difficult. When the weight of an object is lighter than expected, excessive forces are reduced primarily in association with a specific activation of the cerebellar area [11]. Since one of the mechanisms of motor control deficits in DCD is hypothesized to be associated with dysfunction of the cerebellum, children with DCD may experience difficulty when attempting to adapt the parameters of the precision grip to the object’s weight in response to the unexpected light weight of an object [41]. A verification of the adaptive precision grip controls to objects with different weights in children with DCD may clarify this hypothesis.

## 5. Conclusions

In conclusion, children with poor manual dexterity showed increased spatial instabilities (i.e., changes in force direction and object roll) in the precision gripping and lifting of the object, and they had difficulty adjusting to the different weights of the object as shown by the COP trajectory related to grip force. We suspect that spatial instabilities of the precision grip in children with poor manual dexterity were caused by a reduced ability to effectively use feedback information for movement. These impairments may be improved by rehabilitation using stochastic resonance [42,43], which is a phenomenon in which given sensory-subthreshold mechanical noise stimulation to the body, the sensory-motor system is improved by enhanced sensitivity of the tactile sensory systems. Indeed, our previous studies reported that the use of stochastic resonance improved the manual dexterity of children with DCD [44,45]. Together, the past and present findings indicate that a clarification of the spatial instability of a precision grip may offer therapeutic interventions and a new understanding into possible underlying coordination mechanisms of motor clumsiness.

## Figures and Tables

**Table 1:** The children’s characteristics and clinical features.

	Low Manual Dexterity	High Manual Dexterity
Sample	11	55
Age, y	9.00 (±2.30)	9.16 (±1.93)
Sex:		
Male	4	28
Female	7	27
Preferred hand:		
Right	10	53
Left	1	2
M-ABC-2 percentile score	12.45 (±5.02)	70.38 (±21.90)

**Table 2:** Results of the ANOVAs (Weight  ×  Group).

	Mean ± SD				Two-Way ANOVA								
	Low Manual Dexterity		High Manual Dexterity		Weight			Group			Interaction		
	Heavy	Light	Heavy	Light	F	*p*	η_p_^2^	F	*p*	η_p_^2^	F	*p*	η_p_^2^
Mean GF, N	10.94 ± 1.97	4.36 ± 1.26	10.49 ± 1.93	4.29 ± 1.24	273.78	**<0.01**	0.68	0.49	0.49	<0.01	0.25	0.62	<0.01
CV, GF	0.45 ± 0.14	0.38 ± 0.06	0.34 ± 0.04	0.32 ± 0.05	9.83	**<0.01**	0.07	33.66	**<0.01**	0.21	3.54	0.06	0.03
COP trajectory, mm	34.13 ± 9.18	57.78 ± 16.28	27.21 ± 8.94	38.04 ± 13.65	37.73	**<0.01**	0.23	22.56	**<0.01**	0.16	5.22	**<0.05**	0.04
Mean COP-IS, mm	15.83 ± 5.78	16.6 ± 8.04	17.24 ± 5.71	15.6 ± 5.58	0.10	0.75	<0.01	0.02	0.88	<0.01	0.75	0.39	<0.01
Mean COP-ML, mm	8.70 ± 4.44	8.85 ± 5.01	3.99 ± 1.79	3.69 ± 1.62	0.01	0.91	<0.01	70.27	**<0.01**	0.35	0.15	0.70	<0.01
Mean roll deg	3.22 ± 1.52	3.15 ± 1.33	1.04 ± 0.45	1.14 ± 0.44	1.47	0.23	<0.01	163.96	**<0.01**	0.56	0.28	0.60	<0.01
	**Post-Hoc Analysis**												
	**Between Weight**				**Between Group**								
	**Low Manual Dexterity**		**High Manual Dexterity**		**Heavy**		**Light**						
	*p*	d	*p*	d	*p*	d	*p*	d					
COP trajectory, mm	**<0.01**	1.95	**<0.01**	0.89	0.08	0.57	**<0.01**	1.62					

Notes: Degrees of freedom 1 and 2 for F values are 1 and 128. Mean GF: mean grip force, CV GF: coefficient of variation of the grip force, COP trajectory: total trajectory length of the COP, Mean COP-IS: mean COP position in inferior–superior direction, Mean COP-ML: mean COP position in medial–lateral direction, Mean roll: mean degree of roll.

## Data Availability

The data that support the findings of this study are available from the corresponding author upon reasonable request.
